# First Evaluation of Roux-en-Y Gastric Bypass as a Novel Surgical Treatment for Diabetes and Glucose Metabolism Regulation in Cats

**DOI:** 10.3390/vetsci13030272

**Published:** 2026-03-16

**Authors:** Linfeng Li, Guoxiang Yuan, Qianbo Xiong, Wen Hao, Lingchen Yang

**Affiliations:** 1 College of Veterinary Medicine, Hunan Agricultural University, Changsha 410128, China; lflizero@gmail.com (L.L.); bobx972@stu.hunau.edu.cn (Q.X.); whao17@stu.hunau.edu.cn (W.H.); 2 Ringpai Pet Hospital Management Co., Ltd., Tianjin 300457, China

**Keywords:** Roux-en-Y gastric bypass, feline diabetes mellitus, glucose metabolism, insulin resistance, gastrointestinal hormones

## Abstract

Objective: To evaluate the efficacy of Roux-en-Y gastric bypass (RYGB) in improving glucose regulation and metabolic parameters in feline diabetes mellitus (FDM). Methods: FDM was experimentally induced via partial pancreatectomy, splenectomy, and dexamethasone administration. Following insulin stabilization, the RYGB cohort underwent gastric bypass, while the medical management group received glargine insulin. Untreated diabetic controls were monitored for 12 weeks. Blood glucose (GLU), fructosamine (FRU), biochemical profiles, and metabolic hormones were evaluated pre- and post-intervention. Hepatic and pancreatic tissues were collected for histopathological examination. Results: GLU and FRU concentrations in the RYGB group were significantly lower than in diabetic controls (*p* < 0.05), remaining comparable to the insulin-treated group (*p* > 0.05). Alanine aminotransferase (ALT) and aspartate aminotransferase (AST) activities were significantly reduced post-RYGB (*p* < 0.05), closely matching insulin therapy outcomes (*p* > 0.05). Hormonal assays demonstrated decreased gastric inhibitory polypeptide (GIP) and elevated glucagon-like peptide-1 (GLP-1) in RYGB cats. Histopathologically, the RYGB group exhibited attenuated hepatic steatosis and a higher density of pancreatic islet cells with abundant cytoplasm compared to the control groups. Conclusions: RYGB effectively restores glycemic control and metabolic hormone balance in FDM, promoting morphological improvements in pancreatic islets and offering a highly promising alternative therapy for diabetic felines.

## Simple Summary

Feline diabetes is typically managed with lifelong insulin. This study evaluated Roux-en-Y gastric bypass (RYGB) surgery as a novel treatment for feline diabetes. Using an induced diabetic cat model, researchers compared RYGB to standard insulin therapy. Results demonstrated that RYGB successfully normalized blood glucose and fructosamine levels. RYGB also restored metabolic hormones by decreasing GIP and increasing GLP-1. Furthermore, tissue analysis revealed that RYGB reduced liver fat accumulation and promoted morphological improvements in pancreatic islets. Ultimately, RYGB offers a promising alternative therapy with long-term remission potential for diabetic cats.

## 1. Introduction

Feline diabetes mellitus (FDM) is an increasingly prevalent endocrinopathy in veterinary medicine, largely attributed to modern dietary trends and the rising incidence of obesity among domestic cats. Sharing pathophysiological similarities with human type 2 diabetes mellitus (T2DM), FDM is fundamentally characterized by insulin resistance and β-cell dysfunction, which collectively lead to impaired glucose regulation. Clinical hallmarks shared by both conditions include obesity-induced insulin resistance, progressive loss of β-cell mass, and pancreatic amyloid deposition. These shared features not only make FDM a valuable translational model for studying diabetes interventions [1,2], but also suggest that advanced treatments developed for human T2DM, such as gastric bypass surgery, may hold therapeutic promise for feline patients.

Obesity remains the primary risk factor for FDM; notably, obese cats are nearly four times more likely to develop the disease compared to their lean counterparts [3,4]. Underlying mechanisms, including ectopic fat deposition, endoplasmic reticulum stress, and chronic low-grade inflammation, drive insulin resistance and β-cell exhaustion, ultimately culminating in severe dysregulation of glucose metabolism [5,6]. Furthermore, advanced age (particularly over eight years) and gonadectomy—which alters basal metabolic rate and feeding behavior—significantly compound the risk of developing clinical diabetes [7,8,9].

Current therapeutic strategies for FDM focus primarily on symptomatic management rather than achieving definitive disease remission. While dietary modifications, weight management, and exogenous insulin therapy are the mainstays for improving glycemic control, these approaches generally only delay disease progression and mitigate secondary complications [10]. The clinical need for more efficacious therapies has prompted interest in metabolic surgical interventions such as Roux-en-Y gastric bypass (RYGB), a procedure originally pioneered for human bariatric treatment. As a well-established surgery, RYGB not only induces significant weight loss via gastric restriction and malabsorption but also exerts profound improvements in glycemic control among T2DM patients. Clinical studies demonstrate that RYGB frequently leads to the long-term normalization of blood glucose and drastically reduces reliance on hypoglycemic medications, achieving remission rates up to 83.7% in human T2DM cohorts [11,12]. The underlying metabolic benefits are largely attributed to alterations in enteric endocrine signaling, often conceptualized through the “foregut and hindgut hypotheses,” wherein anatomical bypassing of the proximal intestine and rapid nutrient delivery to the distal gut enhance incretin responses, thereby optimizing glucose homeostasis [13,14].

Although RYGB has been extensively validated in human medicine, its application within veterinary practice remains largely unexplored. Given its profound efficacy in resolving human T2DM, investigating the therapeutic potential of RYGB in FDM could introduce a novel treatment paradigm for this challenging condition. In the present study, we established a robust feline diabetes model utilizing partial pancreatectomy and splenectomy, combined with oral dexamethasone administration. Subsequently, we performed RYGB surgery to rigorously evaluate its therapeutic efficacy and metabolic outcomes in comparison to traditional insulin therapy. The findings of this research aim to provide critical translational insights into the utility of RYGB for FDM, potentially laying the groundwork for its future integration into veterinary clinical practice.

## 2. Materials and Methods

### 2.1. Drugs

The following drugs were used in the study: 0.9% sodium chloride injection and 5% glucose injection (Hengfengqiang Biotechnology Co., Ltd., Nantong, China); propofol injection (Guangdong Jiabo Pharmaceutical Co., Ltd., Qingyuan, China); isoflurane (Tianjin Ruipu Biotechnology Co., Ltd., Tianjin, China); dexmedetomidine hydrochloride (Shanghai Zoetis Enterprise Management Co., Ltd., Shanghai, China); and glargine insulin injection (Gan & Lee Pharmaceuticals, Beijing, China).

### 2.2. Assay Kits

ELISA kits for feline insulin, feline C-peptide, feline GIP (glucose-dependent insulinotropic polypeptide), and feline GLP-1 (glucagon-like peptide-1) were obtained from Jiangsu Jingmei Biotechnology Co., Ltd., Yancheng, China.

### 2.3. Experimental Animals

A total of 24 male, neutered domestic cats, aged 4 to 6 years, with a body weight of 4 to 5 kg, and a body condition score (BCS) of 5 based on the 5-point BCS system were used in this study. The specific scoring criteria referenced the [Feline Body Condition Scoring Chart] [15]. All experimental cats underwent physical examination, complete blood count, and blood biochemical tests, with no abnormalities detected. During the experimental period, all cats were housed individually in cages, with free access to commercial cat food and drinking water. All experimental procedures were conducted in strict accordance with the ethical guidelines for the care and use of laboratory animals and were authorized by the Animal Ethics Committee of the Hunan Agricultural University, China (HAUCEC2024-35).

### 2.4. Establishment of the Diabetes Model

The feline diabetes model was established following the method described by Hoenig [16], using partial pancreatectomy combined with splenectomy and daily oral administration of dexamethasone (1.5 mg/cat). 18 cats were randomly selected to undergo the model induction. Blood glucose was measured from the marginal ear veins, and venous blood from the cephalic vein was collected for serum analysis, including fructosamine (FRU) and glucose metabolism-related hormones. After the successful establishment of the diabetes model, liver and pancreatic tissue samples were collected using biopsy forceps for histopathological section examination.

**Figure 1:**
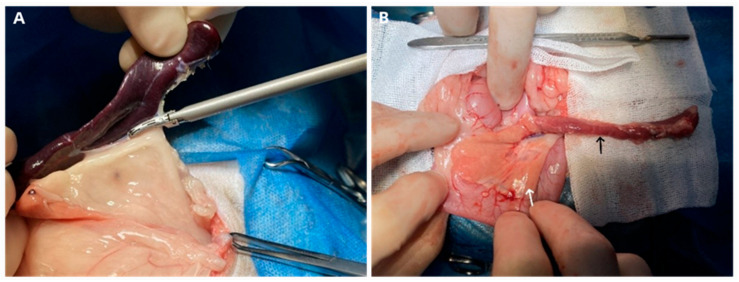
Steps of Pancreatectomy and Splenectomy. (**A**): The spleen is located on the left side of the abdominal cavity and gently pulled out. An ultrasonic scalpel is used to sequentially sever the vessels at the splenic hilum, allowing for the removal of the spleen. (**B**): The left lobe of the pancreas is separated from within the splenorenal ligament. At the junction between the left and right lobes of the pancreas, a 4-0 PDO suture is used to ligate, and then the left lobe of the pancreas is resected. Care is taken to preserve the right lobe of the pancreas and the opening of the pancreatic duct at the major duodenal papilla. The black arrow indicates the left lobe of the pancreas, the white arrow indicates the right lobe, and the dashed line indicates the site of resection of the left lobe.

The surgical procedure involved a midline abdominal incision to access the abdominal cavity. The spleen was removed (Figure 1A), followed by isolation of the left pancreatic lobe (Figure 1B). The pancreatic body connecting the left and right lobes was ligated, and the left lobe was resected, preserving the right lobe and the major pancreatic duct at the duodenal papilla. The abdominal incision was closed with sequential muscle and skin sutures.

### 2.5. Grouping and Treatment of Experimental Animals

Six cats that did not undergo model induction were assigned to the blank control group. The remaining 18 diabetic model cats were randomly divided into three groups: diabetic control, insulin treatment, and gastric bypass. The experiment lasted 12 weeks, starting from week 0. In the diabetic control group, diabetic cats were left untreated for the full 12 weeks to allow symptoms to progress naturally. In the insulin treatment group, insulin was administered from week 1 to week 8, and discontinued from week 9 to week 12. The gastric bypass group also received insulin from week 1 to week 8, underwent RYGB surgery in week 5, and discontinued insulin from week 9 to week 12. Blood glucose levels were measured from the marginal ear veins at weeks 0, 4, 8, and 12. Venous blood was collected from the cephalic veins during the same weeks for FRU and glucose metabolism hormone assays. At week 0 and week 12, liver and pancreatic tissue samples were collected using biopsy forceps for histopathological examination. For cats that died during the experimental period, tissue samples were collected through necropsy.

### 2.6. Roux-en-Y Gastric Bypass Surgery Procedure

**Figure 2:**
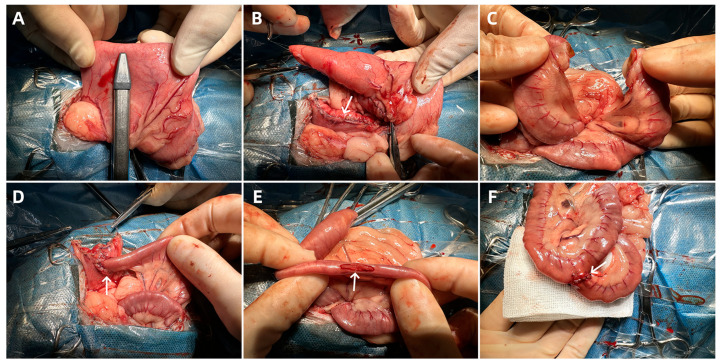
Steps of RYGB. (**A**): Creation of the small gastric pouch. The gastroesophageal junction and greater curvature of the stomach are exposed. A linear cutting stapler is used to create a small gastric pouch extending toward the angle of His. The arrow indicates the angle of His. (**B**): Completion of the small gastric pouch. The staple line is reinforced with continuous and Lembert sutures using 4-0 PDO. The arrow indicates the edge of the newly created small gastric pouch. (**C**): The intestine is transected approximately 10 cm distal to the ligament of Treitz, with a portion of the mesentery also being divided. The proximal end of the intestine becomes the biliopancreatic limb, and the distal end becomes the alimentary limb. (**D**): Gastro-jejunostomy. A 1.5 cm incision is made in the anterior wall of the small gastric pouch. The distal end of the jejunum is brought up and anastomosed to the gastric pouch using interrupted 4-0 PDO sutures. The arrow points to the gastro-jejunostomy site. (**E**): The alimentary limb is measured approximately 20 cm distal to the gastro-jejunostomy site. A 1.5 cm incision is made on the mesenteric border of the jejunum. The arrow indicates the incision on the jejunal wall. (**F**): The biliopancreatic limb (proximal jejunum) is anastomosed to the alimentary limb via a side-to-side jejuno-jejunostomy. Interrupted 4-0 PDO sutures are used, and the mesenteric defect is closed. The arrow indicates the jejuno-jejunostomy site.

The RYGB surgery was performed with the surgeon positioned on the right side of the cat. A midline abdominal incision was made from below the xiphoid process to above the umbilicus. The stomach was exposed by pulling it out through the greater curvature, and approximately 2–3 cm below the cardia, the lesser omentum was dissected to access the posterior gastric space, taking care to avoid damaging the vagal nerve trunks [17]. A linear cutting stapler (Jiangsu Kangji Medical Instrument Co., Ltd., Yancheng, China) was used to create a gastric pouch with a volume of 10–15 mL. The staple line was reinforced with continuous inverting sutures (Figure 2A,B).

A segment of the intestine, approximately 10 cm in length, is measured from the Treitz ligament to the distal end. The intestine is transected at this point, and the mesentery is cut for about 2 cm (Figure 2C). This segment of the intestine will serve as the biliopancreatic limb. A 1.5 cm incision is made on the anterior wall of the small gastric pouch for the gastro-jejunostomy, and the distal end of the jejunum is pulled to this site for the anastomosis (Figure 2D). Approximately 20 cm of intestine is measured distal to the gastro-jejunostomy site, and a 1 cm incision is made on the mesenteric border at this point. This segment of the intestine will serve as the nutrient limb. The biliopancreatic limb, i.e., the proximal jejunum, is pulled to the jejunal side-wall incision for a side-to-side jejuno-jejunostomy, and the mesenteric defect is closed (Figure 2E,F).

### 2.7. Sample Analysis

#### 2.7.1. Blood Glucose Measurement

Blood glucose (GLU) levels were measured using a pet glucose meter (AM10 CA, Jin Hong Medical Material Technology Co., Ltd., Ningbo, China). GLU test strips were inserted into the meter, and approximately 0.5 μL of blood was drawn into the test strip, after which the GLU value was displayed.

#### 2.7.2. FRU and Blood Biochemical Indices Measurement

Fructosamine (FRU) and blood biochemical indices were measured using an automated biochemical analyzer (IDEXX Catalyst One, IDEXX Laboratories, ME). Testing reagent slides, sample cups, and pipette tips were placed into the sample holder, and 100 μL of serum was added to run the test program.

#### 2.7.3. Glucose Metabolism Hormone Measurement

Glucose metabolism hormones were measured using ELISA kits for feline insulin, C-peptide, gastric inhibitory polypeptide (GIP), and glucagon-like peptide-1 (GLP-1). Serum samples (10 μL) were processed according to the manufacturer’s instructions (Jiangsu Jingmei Biotechnology Co., Ltd., Yancheng, China), and absorbance was measured to determine concentrations.

#### 2.7.4. H&E Staining Histopathological Analysis

Tissue samples were fixed in 4% paraformaldehyde, dehydrated through a gradient of ethanol, cleared with xylene, and infiltrated with paraffin. They were then embedded in paraffin to create 4 μm thick paraffin sections. These sections were dewaxed with xylene to water, stained with hematoxylin and eosin, dehydrated, and mounted with neutral gum to produce the final slides.

### 2.8. Data Analysis

Statistical analyses were performed using IBM SPSS Statistics software vision 25. Data were expressed as mean ± standard error of the mean (X ± SEM). One-way analysis of variance (ANOVA) was used for comparison. If variances were homogeneous, LSD tests were applied; otherwise, Tamhane’s T2 test was used. A *p*-value < 0.05 was considered statistically significant. Statistical graphs were created using GraphPad Prism 8.

## 3. Results

### 3.1. Establishment of the Diabetes Model

All 18 cats recovered well following partial pancreatectomy and splenectomy. After oral administration of dexamethasone sodium phosphate, clinical signs such as polydipsia, polyuria, and increased appetite gradually appeared, indicating successful diabetes induction.

**Figure 3:**
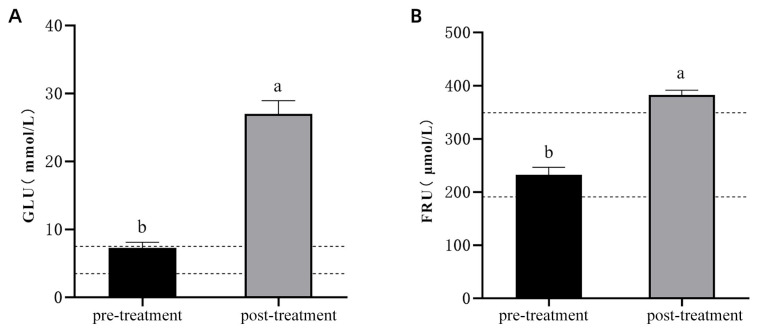
Changes in GLU and FRU Levels Before and After Diabetes Induction in Experimental Cats. (**A**): GLU levels before and after diabetes induction. (**B**): FRU levels before and after diabetes induction. Note: The labels “a,b” indicate significant differences in the comparison of the indicators before and after model induction (*p* < 0.05). The dashed lines represent the upper and lower limits of the normal range for the respective indicators.

GLU levels were significantly higher after model induction compared to pre-model levels (Figure 3A; *p* < 0.05), exceeding the normal feline range of 3.5–7.5 mmol/L. Similarly, FRU levels were also significantly elevated after model induction (Figure 3B; *p* < 0.05), surpassing the normal range of 191–345 μmol/L. These results confirm the successful establishment of the feline diabetes model.

**Figure 4:**
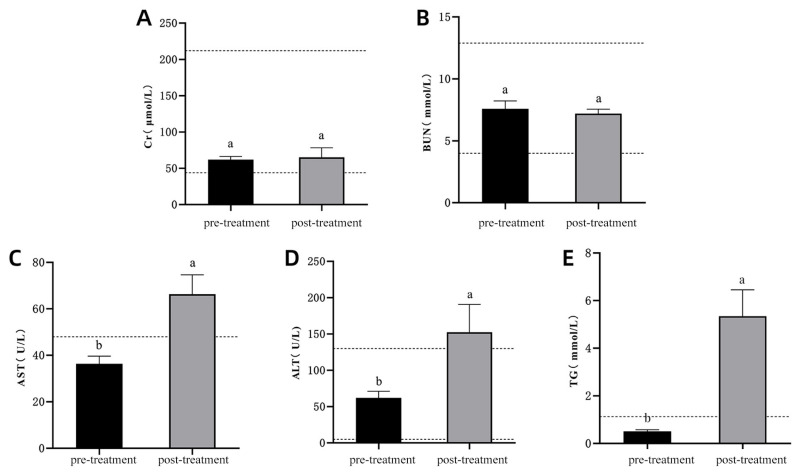
Blood Biochemical Indices Before and After Diabetes Induction in Experimental Cats. (**A**): Changes in Cr levels; (**B**): Changes in BUN levels; (**C**): Changes in AST levels; (**D**): Changes in ALT levels; (**E**): Changes in TG levels. Note: The labels “a,b” indicate significant differences in the comparison of the indicators before and after model induction (*p* < 0.05). The dashed lines represent the upper and lower limits of the normal range for the respective indicators.

There were no significant changes in creatinine (Cr) and blood urea nitrogen (BUN) levels before and after diabetes induction (Figure 4A,B; *p* > 0.05), and both remained within the normal range. In contrast, aspartate aminotransferase (AST), alanine aminotransferase (ALT), and triglycerides (TG) levels were significantly elevated post-induction (Figure 4C–E; *p* < 0.05) and exceeded the normal range, indicating potential liver dysfunction and lipid metabolism disturbances following diabetes induction.

**Figure 5:**
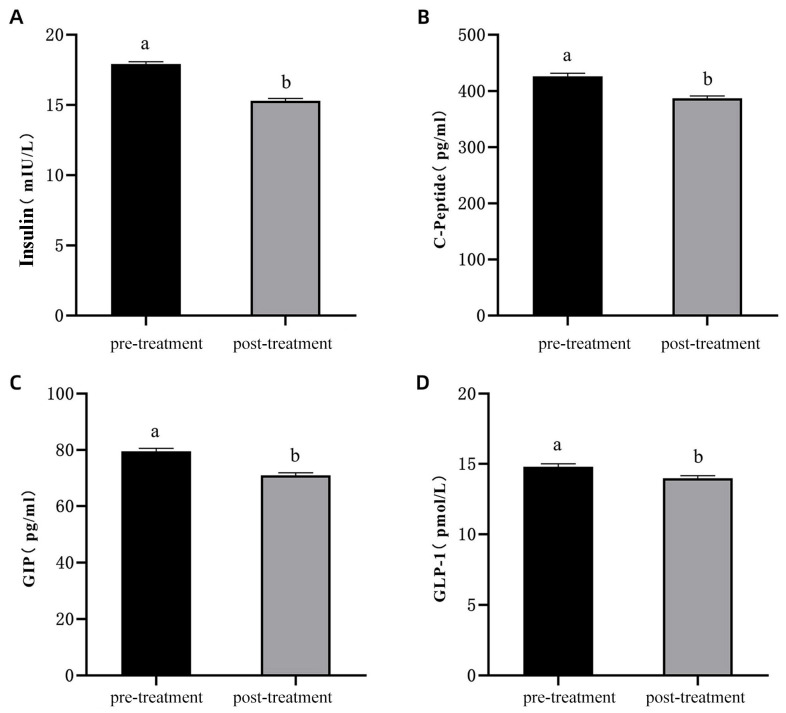
Changes in Glucose Metabolism Hormone Levels Before and After Diabetes Induction in Experimental Cats. (**A**): Changes in insulin resistance levels; (**B**): Changes in C-peptide levels; (**C**): Changes in GIP levels; (**D**): Changes in GLP-1 levels. Note: The labels “a,b” indicate significant differences in the comparison of the indicators before and after model induction (*p* < 0.05).

Serum levels of INS, C-peptide, GIP, and GLP-1 were significantly decreased after diabetes induction compared to pre-induction levels (Figure 5A–D; *p* < 0.05). These changes reflect impaired glucose metabolism following the establishment of the diabetes model.

**Figure 6:**
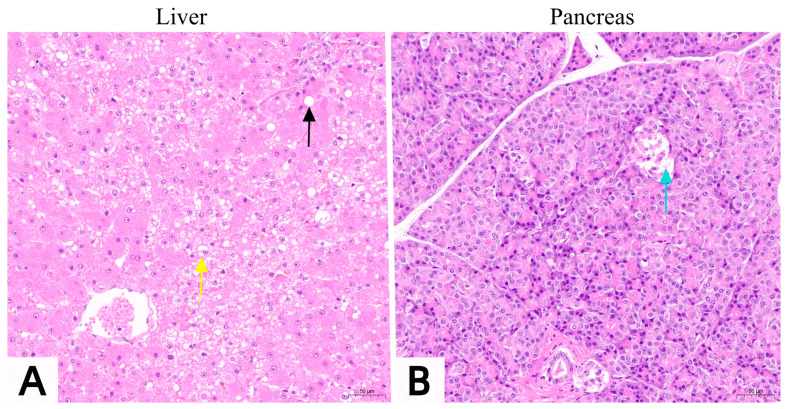
Histological sections of liver and pancreas in experimental cats after diabetes induction. (**A**): Mildly abnormal liver tissue structure with steatosis in hepatocytes, showing numerous variably sized round vacuoles in the cytoplasm (indicated by black arrows), and edema in some hepatocytes (indicated by yellow arrows); uniform size of hepatic sinusoids without dilation, tightly arranged hepatic cords; no inflammatory cell infiltration in the hepatic parenchyma. (**B**): Mildly abnormal pancreatic tissue structure with edema in some islet cells and a reduced number of islet cells (indicated by blue arrows); no inflammatory cell infiltration within the tissue.

After the successful establishment of the diabetes model, hepatic steatosis occurred in hepatocytes (Figure 6A), and some islet cells showed edema and a reduced number (Figure 6B). These pathological changes indicate that the establishment of the diabetes model leads to fatty liver and islet cell damage.

### 3.2. Evaluation of Roux-en-Y Gastric Bypass in Feline Diabetes

#### 3.2.1. Clinical Observations

In the blank control group, no adverse symptoms were observed throughout the experiment. Cats in the insulin treatment group maintained good condition during the 8-week treatment. However, after discontinuing insulin in week 9, symptoms such as polydipsia, polyuria, and increased appetite reappeared. In the gastric bypass group, cats experienced poor appetite and mental status for the first 3 days post-surgery, but gradually recovered with supportive care. By 2 weeks post-surgery, they resumed normal eating, with reduced food intake and significant short-term weight loss, though weight gradually increased post-recovery. In contrast, the diabetic control group exhibited severe symptoms such as polydipsia, polyuria, weight loss, and lethargy. By week 8, these cats showed jaundice, vomiting, and entered a comatose state. At this time, immediate treatment measures were taken for these cats, but they still died within 3 to 5 days. No data were available for this group after week 8.

#### 3.2.2. Blood Glucose and Fructosamine Levels

**Figure 7:**
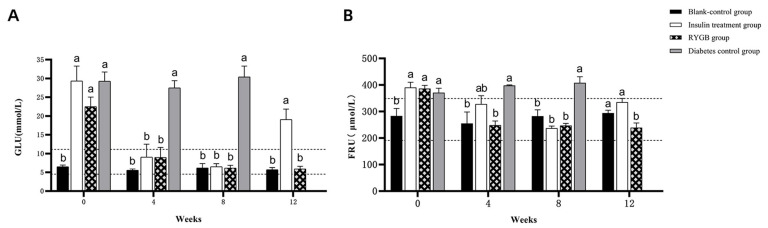
Blood Glucose and FRU Levels in Experimental Cats. (**A**): GLU levels at different time points. (**B**): FRU levels at different time points. Note: The labels “a,b” indicate significant differences in the comparison of the same indicator between different groups at the same time point (*p* < 0.05). The dashed lines represent the upper and lower limits of the normal range for the respective indicators.

At week 0, GLU levels in the insulin, gastric bypass, and diabetic control groups were significantly higher than in the blank control group (*p* < 0.05), exceeding the normal range (3.5–7.5 mmol/L). From weeks 4 to 8, blood glucose levels in the insulin and gastric bypass groups normalized, while levels in the diabetic group remained elevated. By week 12, blood glucose levels in the gastric bypass group were within the normal range and comparable to the blank control group (*p* > 0.05), while the insulin group’s levels were still elevated (Figure 7A).

Serum FRU levels followed a similar pattern. In week 0, FRU levels in the insulin, gastric bypass, and diabetic groups were significantly higher than in the blank control group (*p* < 0.05). By weeks 4 and 8, FRU levels in the insulin and gastric bypass groups returned to normal, while levels in the diabetic group remained elevated. By week 12, FRU levels in the gastric bypass group were significantly lower than in the insulin group and remained within the normal range (Figure 7B).

#### 3.2.3. Changes in Blood Biochemical Indices

**Figure 8:**
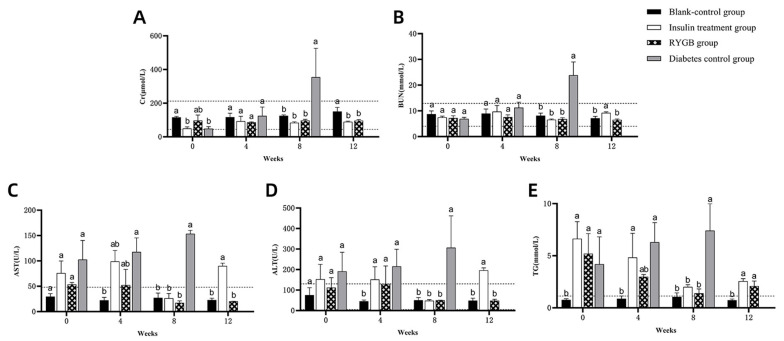
Changes in Blood Biochemical Indices in Experimental Cats. (**A**): Cr levels across all groups (weeks 0 to 12); (**B**): BUN levels across all groups (weeks 0 to 12); (**C**): AST levels across all groups (weeks 0 to 12); (**D**): ALT levels across all groups (weeks 0 to 12); (**E**): TG levels across all groups (weeks 0 to 12). Note: The labels “a,b” indicate significant differences in the comparison of the same indicator between different groups at the same time point (*p* < 0.05). The dashed lines represent the upper and lower limits of the normal range for the respective indicators.

The comparisons of Cr, BUN, AST, ALT, and TG levels at different time points are summarized as follows:

At weeks 0 and 4, serum Cr levels in all groups remained within the normal range (44.0–212.0 μmol/L) with no significant differences (*p* > 0.05). By week 8, the diabetic group exhibited significantly elevated Cr levels (*p* < 0.05), while in week 12, Cr levels in all groups were within the normal range (Figure 8A).

BUN levels remained within the normal range (4.00–12.90 mmol/L) for all groups up to week 4. At week 8, BUN levels in the diabetic group were significantly elevated (*p* < 0.05), but by week 12, the gastric bypass and blank control groups had similar BUN levels within the normal range (Figure 8B; *p* > 0.05).

At week 0, serum AST levels in all groups exceeded the normal range (0–48 U/L). By week 8, AST levels in the diabetic group remained significantly higher than in other groups (*p* < 0.05), while the other groups had returned to normal. By week 12, AST levels in the gastric bypass and blank control groups were within the normal range (Figure 8C).

Serum ALT levels in the insulin treatment and diabetic groups were elevated at week 0, exceeding the normal range (5–130 U/L). By week 4, the gastric bypass and insulin treatment groups had significantly elevated ALT levels (*p* < 0.05). By week 12, ALT levels in the gastric bypass group normalized, while levels in the insulin treatment group remained elevated (Figure 8D).

TG levels in the insulin treatment, gastric bypass, and diabetic groups were higher than in the blank control group at week 0 (*p* < 0.05). By week 8, TG levels in the insulin and gastric bypass groups decreased, while the diabetic group remained elevated. By week 12, TG levels in the insulin and gastric bypass groups remained elevated compared to the blank control group but were closer to normal levels (Figure 8E).

#### 3.2.4. Changes in Glucose Metabolism Hormones

**Figure 9:**
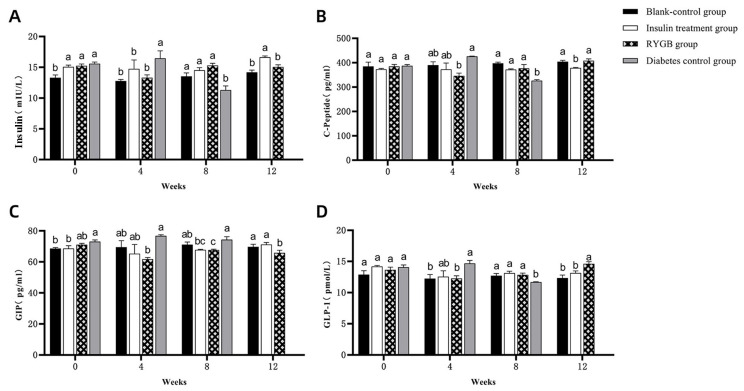
Glucose Metabolism Hormone Indices in Experimental Cats. (**A**): Comparison of insulin resistance levels in the blank control group, insulin treatment group, gastric bypass group, and diabetic group from weeks 0 to 12. (**B**): Comparison of C-peptide levels in the blank control group, insulin treatment group, gastric bypass group, and diabetic group from weeks 0 to 12. (**C**): Comparison of GIP levels in the blank control group, insulin treatment group, gastric bypass group, and diabetic group from weeks 0 to 12. (**D**): Comparison of GLP-1 levels in the blank control group, insulin treatment group, gastric bypass group, and diabetic group from weeks 0 to 12. Note: The labels “a,b” indicate significant differences in the comparison of the same indicator between different groups at the same time point (*p* < 0.05).

INS levels (Figure 9A) in the blank control group were significantly lower than those in the insulin treatment, gastric bypass, and diabetic groups at week 0 (*p* < 0.05), with no significant differences among the latter three groups (*p* > 0.05). By week 4, insulin levels in the diabetic group were significantly higher than in the other groups (*p* < 0.05), indicating increased insulin resistance, while no significant differences were observed between the blank control, insulin treatment, and gastric bypass groups (*p* > 0.05). By week 8, insulin levels in the diabetic group were significantly lower than in the other groups (*p* < 0.05), suggesting β-cell exhaustion. In week 12, insulin levels in the insulin treatment group were significantly higher than those in the gastric bypass and blank control groups (*p* < 0.05), while no significant differences were seen between the gastric bypass group and the blank control group (*p* > 0.05). This indicates that the gastric bypass group maintains insulin levels comparable to healthy cats, highlighting the potential of the surgery to improve insulin sensitivity and stabilize insulin secretion.

C-peptide, a marker of endogenous insulin secretion, exhibited similar trends (Figure 9B). At week 0, no significant differences were observed among the groups (*p* > 0.05). By week 4, the diabetic group had significantly higher C-peptide levels compared to the gastric bypass group (*p* < 0.05), reflecting higher insulin secretion due to increased resistance. By week 8, the diabetic group’s C-peptide levels dropped significantly compared to other groups (*p* < 0.05), indicating β-cell dysfunction. By week 12, C-peptide levels in the insulin treatment group were significantly lower than in the gastric bypass and blank control groups (*p* < 0.05), while the gastric bypass group maintained similar levels to the blank control group, suggesting restored β-cell function post-surgery.

The comparison of GIP levels (Figure 9C) showed that at week 0, GIP levels in the diabetic group were significantly higher than in the blank control group (*p* < 0.05), reflecting elevated insulinotropic stimulus. By week 4, GIP levels in the gastric bypass group were significantly lower than in the diabetic group (*p* < 0.05), but similar to the blank control and insulin treatment groups (*p* > 0.05). By week 8, GIP levels in the gastric bypass group were significantly lower than in the blank control and diabetic groups (*p* < 0.05), indicating reduced stimulus for insulin secretion post-surgery. By week 12, GIP levels in the gastric bypass group were significantly lower than in the blank control and insulin treatment groups (*p* < 0.05), further supporting the role of gastric bypass in normalizing GIP levels and improving glucose metabolism.

GLP-1 levels, another important incretin hormone, also showed distinct patterns (Figure 9D). At week 0, no significant differences were observed among the groups (*p* > 0.05). By week 4, the diabetic group had significantly higher GLP-1 levels compared to the gastric bypass and blank control groups (*p* < 0.05), possibly reflecting compensatory mechanisms. However, by week 8, GLP-1 levels in the gastric bypass, insulin treatment, and blank control groups were significantly higher than in the diabetic group (*p* < 0.05), indicating improved incretin function in these groups. By week 12, the gastric bypass group had significantly higher GLP-1 levels compared to the blank control and insulin treatment groups (*p* < 0.05), suggesting enhanced incretin activity after surgery, which could contribute to better postprandial glucose control and improved insulin sensitivity.

#### 3.2.5. Histopathological Changes in Liver and Pancreas

**Figure 10:**
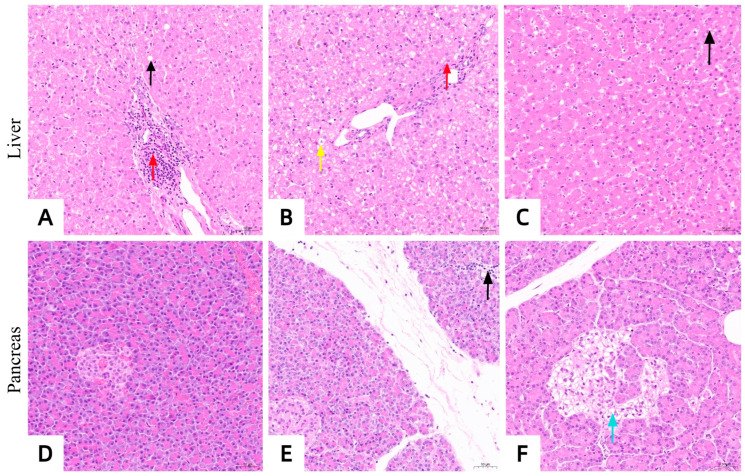
Histopathological sections of liver and Pancreas tissues. (**A**): Liver in insulin treatment group; The liver tissue structure was slightly abnormal, with mild steatosis of hepatocytes in the field of vision, and several round vacuoles in the cytoplasm were seen, as indicated by the black arrow. The hepatic sinusoids were uniform in size without dilatation, and the hepatic cords were closely arranged. A partial inflammatory cell infiltration was seen in the liver parenchyma, as indicated by the red arrow. (**B**): Liver in RYGB group; The liver tissue structure was slightly abnormal, and some hepatocytes were edematous in the visual field, as indicated by the yellow arrow. The hepatic sinusoids were uniform in size without dilatation, and the hepatic cords were closely arranged. Inflammatory cell infiltration was observed in the liver parenchyma, as indicated by the red arrow. (**C**): Liver in diabetes control group; The liver tissue structure was slightly abnormal, with steatosis of hepatocytes in the field of vision, and several round vacuoles in the cytoplasm were seen, as indicated by the black arrow. The hepatic sinusoids were uniform in size without dilatation, and the hepatic cords were closely arranged. No inflammatory cell infiltration was observed in the liver parenchyma. (**D**): Pancreas in insulin treatment group; The pancreatic tissue structure was basically normal, and the islets were spherical cell clusters, distributed between acini, with clear boundaries with the surrounding glands. The cells in the islets were arranged regularly, with high cell density and abundant cytoplasm, without telangiectasia. No inflammatory cell infiltration was observed in the tissues. (**E**): Pancreas in RYGB group; The pancreatic tissue structure was slightly abnormal, and the islets were spherical cell clump-like structure, distributed between the acini, with clear boundaries with the surrounding glands. The cells in the islets were arranged regularly, with high cell density and abundant cytoplasm, and no telangiectasia was observed. A small amount of inflammatory cell infiltration was observed within the tissue, as indicated by the black arrow. (**F**): Pancreas of diabetes control group; The pancreatic tissue structure was slightly abnormal, and some islet cells in the field of vision were edema and the number was reduced, as shown by the blue arrow. No inflammatory cell infiltration was observed in the tissues.

At week 12, the liver tissue pathological sections of the insulin treatment group, the gastric bypass surgery group, and the diabetic control group are shown in Figure 10A–C. The liver tissue sections of the insulin treatment group (Figure 10A) showed mild steatosis of liver cells and partial infiltration of inflammatory cells in the liver parenchyma. The liver tissue sections of the gastric bypass surgery group (Figure 10B) showed mild edema of some hepatocytes, uniform size of hepatic sinusoids, closely arranged hepatic cords, and inflammatory cell infiltration in the liver parenchyma, but no steatosis occurred. The liver tissue sections of the diabetic control group (Figure 10C) showed steatosis of the liver tissue without inflammatory cell infiltration. The degree of steatosis was more severe compared with the insulin treatment group.

At week 12, the pathological sections of pancreatic tissues in the insulin treatment group, the gastric bypass surgery group, and the diabetic control group are shown in Figure 10D–F. The pancreatic tissue sections of the insulin treatment group (Figure 10D) showed that the cells in the islets were arranged regularly, with a high cell density, abundant cytoplasm, and no inflammatory cell infiltration. The pancreatic tissue sections of the gastric bypass surgery group (Figure 10E)

Showed a spherical cell mass structure of the islets, distributed between the acini, with a clear boundary from the surrounding glands. The cells within the islets were arranged regularly, with a high cell density and abundant cytoplasm. However, a small amount of inflammatory cells infiltrated the tissue. The pancreatic tissue sections of the diabetic control group (Figure 10F) showed abnormal pancreatic tissue structure, with edema of some islet cells and no infiltration of inflammatory cells. Compared with the insulin treatment group and the gastric bypass surgery group, the number of islet cells was significantly reduced.

## 4. Discussion

In this study, a robust feline diabetes model was successfully established via partial pancreatectomy, splenectomy, and dexamethasone administration. While the concurrent splenectomy was anatomically necessitated to safely resect the left pancreatic lobe, it introduces a potential confounding factor. Although the spleen is primarily an immune organ, its absence might subtly alter the systemic inflammatory and metabolic milieu, which should be considered when interpreting the overall metabolic outcomes. This model provided the physiological milieu necessary to evaluate the therapeutic efficacy of RYGB surgery in managing diabetes. The induced condition closely mirrored human T2DM, characterized by primary insulin resistance and progressive β-cell dysfunction. Consequently, this model offered a reliable platform to investigate the underlying mechanisms of RYGB and its potential to alleviate diabetic clinical signs.

The successful induction of diabetes was confirmed by sustained hyperglycemia, evidenced by elevated blood glucose and fructosamine (FRU) concentrations. FRU, a biomarker reflecting average glycemic control over the preceding 2–3 weeks, confirmed prolonged post-induction hyperglycemia [18]. Concurrent elevations in serum ALT and AST activities, alongside increased TG concentrations, suggested early-stage hepatic dysfunction—a frequent comorbidity of diabetes, particularly feline hepatic lipidosis [19]. Notably, serum Cr and BUN concentrations remained within normal reference intervals, indicating that renal function had not yet been compromised and aligning with early-stage diabetic progression. Furthermore, histopathological evidence of hepatic steatosis and reduced islet cell density was consistent with the pathophysiological hallmarks of T2DM, corroborating the hematological findings. These hepatic and pancreatic alterations closely mimic early-stage T2DM in humans, further validating the translational efficacy of this feline model.

A paramount finding of this study was the capacity of RYGB to maintain normoglycemia independent of exogenous insulin therapy. By week 12, blood glucose and FRU concentrations in the RYGB cohort stabilized within normal reference intervals, despite the cessation of insulin therapy for four weeks. Conversely, the insulin-treated group exhibited rebound hyperglycemia following insulin withdrawal. This underscores the superior potential of RYGB to provide sustained glycemic control by ameliorating insulin resistance and partially restoring β-cell function [20]. This durability was starkly contrasted by the diabetic control group, which succumbed to severe metabolic derangements and multiorgan failure by week 8 [21]. These outcomes highlight the clinical viability of RYGB as a more durable therapeutic intervention than conventional insulin therapy. Consistent with human T2DM data, RYGB significantly mitigated diabetic clinical signs and reduced or eliminated exogenous insulin dependence, suggesting conserved therapeutic mechanisms across mammalian species.

Beyond glycemic control, RYGB conferred significant benefits regarding hepatic function and lipid metabolism. The normalization of ALT and AST activities by 8 weeks post-surgery suggests that RYGB reversed diabetes-associated hepatopathy, likely secondary to improved glycemic regulation and lipid partitioning [22]. Although TG concentrations in the RYGB group remained mildly elevated above reference intervals, they were significantly reduced from preoperative baseline levels, implying that sustained glycemic stability may ultimately normalize lipid profiles over time. Conversely, insulin therapy yielded only transient hepatic improvements; ALT and AST activities rebounded upon insulin discontinuation, indicating that exogenous insulin alone is insufficient for long-term metabolic restoration.

The endocrine alterations observed herein provide mechanistic insights into the therapeutic efficacy of RYGB. Preoperatively elevated GIP concentrations decreased significantly post-RYGB, whereas GLP-1 concentrations increased, particularly by week 12. These hormonal shifts substantiate the “foregut” and “hindgut” hypotheses regarding RYGB-mediated glycemic control [23]. The foregut hypothesis posits that bypassing the proximal small intestine attenuates the secretion of anti-incretin signals such as GIP, while the hindgut hypothesis suggests that the accelerated delivery of nutrients to the distal intestine stimulates GLP-1 secretion, thereby enhancing insulin sensitivity and β-cell function [24]. The temporal dynamics of these changes—with GIP declining by week 4 and GLP-1 peaking by week 12—suggest that GIP reduction may serve as an early biomarker of surgical efficacy, whereas GLP-1 elevation drives long-term glycemic homeostasis [25,26]. These findings parallel human T2DM literature, wherein RYGB-induced modulation of incretin hormones mitigates insulin resistance and enhances β-cell function.

Postoperative stabilization of endogenous insulin and C-peptide concentrations in the RYGB group further supports the premise that this surgery primarily ameliorates peripheral insulin resistance rather than directly upregulating insulin hypersecretion [27]. Unlike the insulin-treated cohort, which experienced hormonal fluctuations corresponding to exogenous dosing, the RYGB group maintained physiological insulinemic stability. This is clinically pivotal, as it addresses the core pathophysiology of diabetes—insulin resistance—rather than merely superseding β-cell exhaustion with exogenous hormone replacement [28,29]. By restoring endogenous insulin sensitivity and secretory capacity, RYGB provides a comprehensive metabolic intervention capable of inducing clinical remission without the requirement for lifelong insulin administration.

The prophylactic potential of RYGB against progressive diabetic complications was also demonstrated. While renal biomarkers (Cr and BUN) remained stable in both treatment groups, they progressively elevated in the diabetic controls, indicative of deteriorating renal function [30]. This highlights the necessity of early glycemic intervention to prevent diabetic nephropathy, a ubiquitous sequela of unmanaged diabetes [31]. Furthermore, the postoperative improvements in hepatic parameters and lipid metabolism suggest that RYGB may protect against secondary comorbidities, including hepatic lipidosis and cardiovascular dysfunction [32]. Conclusively, RYGB not only resolves hyperglycemia but also interrupts the cascade of secondary metabolic derangements that drive disease progression.

Histopathological evaluations of hepatic and pancreatic tissues further differentiated the treatment modalities. In the insulin-treated group, the presence of mild hepatic steatosis and focal inflammatory infiltrates indicated that while glycemic control was achieved, lipid metabolism remained inadequately addressed in the short term. The pancreatic islets in this group retained regular architecture and high cellular density, reflecting functional preservation directly attributable to external glucose regulation. Predictably, the diabetic control group exhibited severe hepatic steatosis—a fatal, late-stage complication of FDM. This unchecked hyperglycemia, compounded by severe insulin resistance and dyslipidemia, led to extensive islet architectural destruction, characterized by marked cellular edema and depletion. This perpetuates a vicious cycle of metabolic decompensation.

Conversely, the RYGB cohort displayed only mild hepatocellular edema and minor inflammatory infiltration, likely reflecting residual postoperative stress. Notably, significant hepatic steatosis was absent, signifying the profound efficacy of RYGB in correcting hepatic lipid metabolism via improved systemic insulin sensitivity. Furthermore, RYGB-treated cats exhibited robust pancreatic islets with dense, regular cellular arrangements and abundant cytoplasm. These morphological improvements suggest that RYGB-induced incretin modulation fosters a microenvironment conducive to morphological improvement and functional recovery. Collectively, these histopathological findings closely mirror the hepatic lipidosis resolution and structural islet improvements documented in human T2DM patients post-RYGB, mechanistically validating its therapeutic application in feline veterinary practice.

However, several limitations of this study must be acknowledged. Primarily, the surgically induced diabetes model utilized herein may not entirely capture the pathophysiological complexity of naturally occurring feline diabetes, which is a chronic, multifactorial disease heavily influenced by long-term obesity and islet amyloid deposition. Consequently, how these specific metabolic responses translate to spontaneous feline diabetes requires cautious interpretation. Furthermore, although we observed promising morphological improvements in the pancreatic islets post-RYGB, our histological evaluation relied solely on H&E staining without immunohistochemical confirmation of β-cell identity or proliferation. Therefore, our conclusions regarding islet structural preservation must be moderated, and further molecular studies—such as those utilizing β-cell specific markers and proliferation assays—are needed to definitively confirm cellular regeneration and repair.

## 5. Conclusions

This study demonstrates that RYGB is a highly effective intervention for managing feline diabetes in a surgically induced model, with the potential to induce long-term remission without the need for exogenous insulin. The surgery’s ability to improve insulin sensitivity, stabilize blood glucose, promote morphological improvements in pancreatic islets, and restore metabolic hormones offers a promising therapeutic approach for diabetes management. Moreover, the significant improvements in liver function and lipid metabolism highlight RYGB’s potential to prevent the complications of diabetes, improving both metabolic outcomes and quality of life. These findings have important implications not only for veterinary medicine but also for broader diabetes research, where RYGB could serve as an alternative to conventional insulin-based therapies. Future research should explore the long-term effects of RYGB, incorporate detailed dietary tracking and molecular β-cell evaluations, assess its efficacy in naturally occurring feline diabetes, and investigate its role in preventing the progression of diabetic complications.

## Data Availability

The original contributions presented in this study are included in this article. Further inquiries can be directed to the corresponding author.
